# Case Report: CNNM2 Mutations Cause Damaged Brain Development and Intractable Epilepsy in a Patient Without Hypomagnesemia

**DOI:** 10.3389/fgene.2021.705734

**Published:** 2021-08-20

**Authors:** Xiucui Li, Shijia Bao, Wei Wang, Xulai Shi, Ying Hu, Feng Li, Qianlei Zhao, Feixia Zheng, Zhongdong Lin

**Affiliations:** ^1^Department of Pediatric Neurology, The Second Affiliated Hospital and Yuying Children's Hospital of Wenzhou Medical University, Wenzhou, China; ^2^Department of Clinical Medicine, Wenzhou Medical University, Wenzhou, China; ^3^Department of Pediatric Orthopedics, The Second Affiliated Hospital and Yuying Children's Hospital of Wenzhou Medical University, Wenzhou, China

**Keywords:** CNNM2, CNBH domain, hypomagnesemia, intellectual disability, intractable epilepsy

## Abstract

A series of neurological manifestations such as intellectual disability and epilepsy are closely related to hypomagnesemia. Cyclin M2 (CNNM2) proteins, as a member of magnesium (Mg^2+^) transporters, were found along the basolateral membrane of distal renal tubules and involved in the reabsorption of Mg^2+^. Homozygous and heterozygous variants in *CNNM2* reported so far were responsible for a variable degree of hypomagnesemia, several of which also showed varying degrees of neurological phenotypes such as intellectual disability and epilepsy. Here, we report a *de novo* heterozygous *CNNM2* variant (c.2228C > T, p.Ser743Phe) in a Chinese patient, which is the variant located in the cyclic nucleotide monophosphate-binding homology (CNBH) domain of CNNM2 proteins. The patient presented with mild intellectual disability and refractory epilepsy but without hypomagnesemia. Thus, we reviewed the literature and analyzed the phenotypes related to *CNNM2* variants, and then concluded that the number of variant alleles and the changed protein domains correlates with the severity of the disease, and speculated that the CNBH domain of CNNM2 possibly plays a limited role in Mg^2+^ transport but a significant role in brain development. Furthermore, it can be speculated that neurological phenotypes such as intellectual disability and seizures can be purely caused by CNNM2 variants.

## Introduction

As an abundant intracellular divalent cation in human body, magnesium (Mg^2+^) plays an important role in numerous biological processes such as the synthesis of RNA, DNA and protein, and the production and storage of cellular energy (Volpe, 2013). Transmembrane transport of Mg^2+^ requires the action of specialized proteins known as Mg^2+^ transporters. In mammals, eight diverse sorts of Mg^2+^ transporters have been identified so far, many of which are related to numerous congenital diseases such as neural tube defects (Walder et al., 2009) and spastic paraplegia (Rainier et al., 2003), involving a diversity of tissues, most prominently in intestine, kidney, brain and skin (Quamme, 2010). Cyclin M2 (CNNM2) is one of the members of the Mg^2+^ transporters, participating in Mg^2+^ reabsorption in kidney tubules.

Variants in *CNNM2* gene (MIM 607803) had been related to multiple phenotypes, ranging from single hypomagnesemia to severe intellectual disability and intractable epilepsy. Evidence has been provided that heterozygous variants in *CNNM2* gene can cause renal hypomagnesemia (HOMG6 [MIM 613882]) (Stuiver et al., 2011), seizures, and intellectual disability (HOMGSMR1 [MIM 616418]). Among them, HOMGSMR1 is characterized by onset of seizures related to hypomagnesemia in the first year of life and affected individuals show delayed psychomotor development in variable degrees (Arjona et al., 2014). Heterozygous variants in *CNNM2* gene were described for the first time in two unrelated families with autosomal dominant renal hypomagnesemia, displaying severely lowered serum Mg^2+^ levels and a defect in tubular Mg^2+^ reabsorption, without other electrolyte disturbances or neurological function impairment (Stuiver et al., 2011). Subsequently, the other four heterozygous and two homozygous variants in *CNNM2* gene were reported to cause patients variable degrees of intellectual disability except for autosomal dominant or recessive renal hypomagnesemia, and most of whom had seizures(Arjona et al., 2014; Accogli et al., 2019; Bamhraz et al., 2020). What is noteworthy is that all reported cases showed the varying degrees of hypomagnesemia up to now, with or without neurological impairment.

Here, we present a *de novo* heterozygous variant in *CNNM2* in a Chinese girl, presenting with intellectual disability and intractable epilepsy, but no hypomagnesemia.

## Case Description

**Figure 1:**
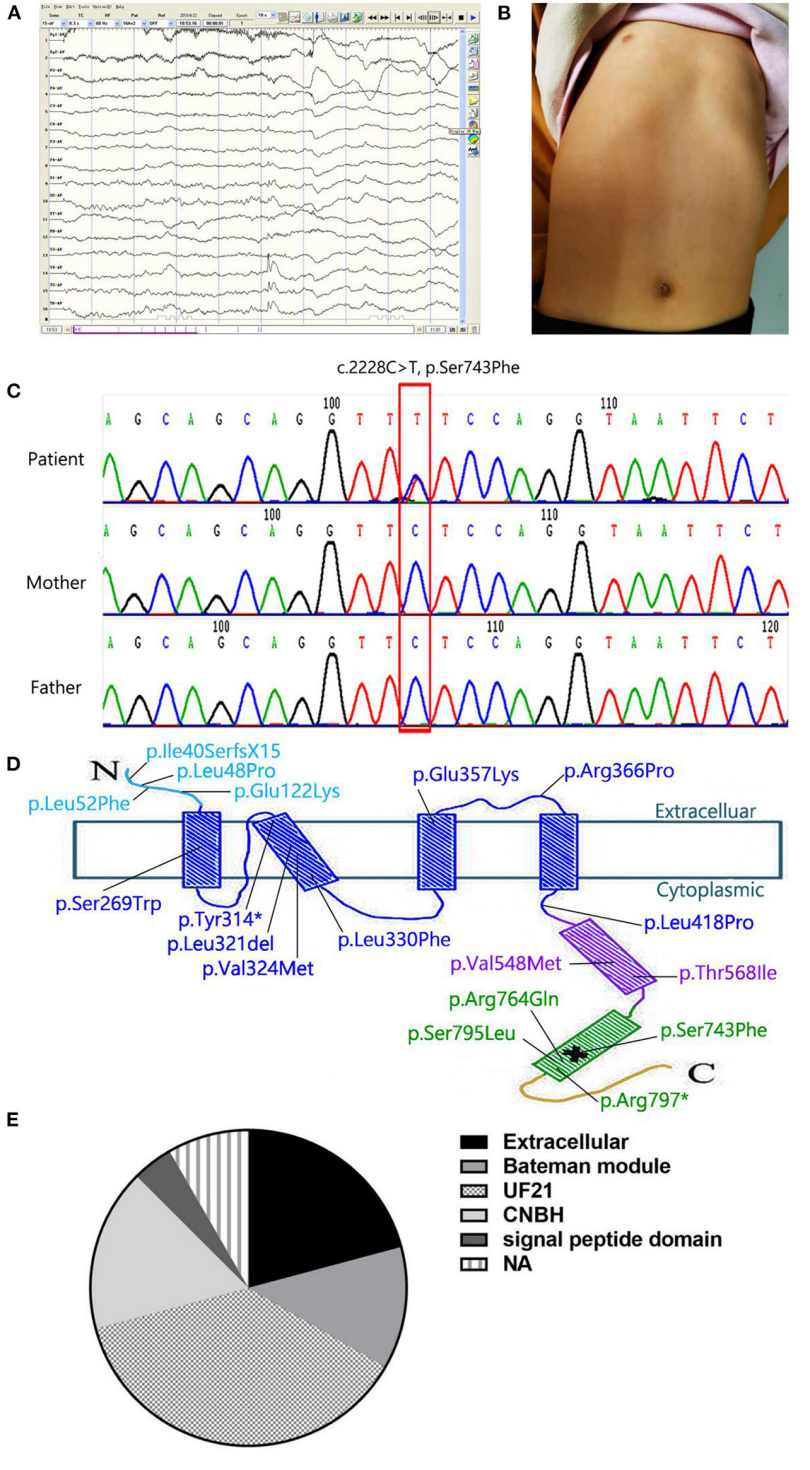
**(A)** The EEG monitoring showed the release of sharp-slow and spinous-slow waves in the left posterior temporal region and the right middle posterior temporal regions. **(B)** Patient's picture showing a funnel-shaped chest. **(C)** Partial *CNNM2* electropherograms of the patient and her parents. In the electropherograms, the variant is indicated by a red box and the changes in nucleotide and resulting effects on the protein are shown. **(D)** Localization of the variant in the secondary structure of CNNM2. The N-terminal extracellular domain and the transmembrane domain are in light blue and dark blue respectively. The CBS domain is in purple, the CNBH domain is in green, and the unstructured C-terminus is yellow. *means stop codon. The location of pathological variant is indicated by a cross. **(E)** variant domains of cases listed in Table 1. In 24 cases, we found 5 domains: extracellular (5/24), bateman module (3/24), UF21(9/24), CNBH(4/24), and signal peptide(1/24). The other two cases were not available (2/24).

A 4-year-old girl attracted our attention with the symptoms of intractable epilepsy and mild intellectual disability. She was found to suffer cerebral convulsions during sleep, characterized by focal seizures since she was 3 years old. The EEG monitoring showed the release of sharp-slow and spinous-slow waves in the left posterior temporal region and the right middle posterior temporal regions (Figure 1A). Despite three kinds of antiepileptic medications (Oxcarbazepine, Valproic acid, Topiramate) were used in proper sequence, the seizures were not controlled, once every two days to half a month. When Lacosamide, the fourth antiepileptic drug, was used, the patient still had focal seizures once every month. She was assessed to be well below age expectations in verbal and social communication skills, and obtained an intelligence quotient score of 68. In physical examination, she had a funnel-shaped chest (Figure 1B). Her head circumference was measured 48 cm, below age expectations, but not diagnosed microcephaly. Other physical examinations were normal. Laboratory investigations demonstrated serum magnesium (0.74 mmol/l, range 0.67 to 1.04 mmol/L) is in the normal range. Declined calcitonin (1.82 pg/ml, range 5.17 to 9.82 pg/ml), and calcium (2.16 mmol/l, range 2.23 to 2.80 mmol/L) were found, and serum phosphorus, parathyroid hormone, and bone specific alkaline phosphatase were normal. We asked the medical history carefully, found that children with respiratory tract infection disease almost every month, resulting in poor appetite, less nutrients intake. She was the only patient in her family. Both parents are healthy, without family history of seizures. Umbilical cord around the neck and meconium stained amniotic fluid occurred when she was in perinatal period, delayed crying and lack of oxygen at birth. Soon afterwards she was found to be delayed in athletic and intellectual development, characterized by a delay in walking and speaking. A cranial magnetic resonance imaging (MRI) at 2 and 5 years of age showed no obvious abnormality.

During follow-up, the patient had focal seizures once every month treated by Oxcarbazepine, Valproic acid, Topiramate and Lacosamide at 7 years old with weight was 14 kg (< −3 SD), height of 105 cm (< −3 SD) and BMI of 12.7 kg/cm^2^. She showed poor social interaction and verbal communication skills. Laboratory investigations demonstrated serum magnesium (0.78 mmol/l, range 0.75 to 1.02 mmol/L) was still in the normal range. Her father and mother had been tested for serum magnesium in our hospital, and serum magnesium was normal, respectively 0.95 mmol/L and 1.03 mmol/L. Uric calcium (3.2 mmol/24 h, range from 2.5 to 7.5 mmol/24 h) in the patient was normal. Urine sodium, potassium and chloride for 24 h were also normal, and 25 hydroxyvitamin D was low (21.04 ng/ml, normal range > = 30). Hypocalcemia may be caused by a lack of nutrients intake.

## Genetic Testing

As an etiological investigation, whole Exome Sequencing was performed for the patient and generated about 10 Gb high-quality raw sequencing data. The average sequencing depth for the sample was 106.98-fold, with 94.48% of coverage of the targeted regions at a 20-fold sequencing depth and 78.95% at 50-fold depth. After removal of sequencing adapters, low-quality reads and duplicated reads, we identified more than 40,000 single nucleotide variations (SNVs) and indels using the GATK tool. Afterwards a series of bioinformatics filtering strategy, including variant filtration against multiple databases, functional prediction by multiple *in silico* tools and gene function, were carried out as described previously (PMID: 30488659, 28386848, 22595939). Finally, three candidate variants were submitted for validation of sanger sequencing from the proband and parents, and only one rare and novel missense variant (c.2228C>T, p.Ser743Phe) in CNNM2 was retain because of the other two variants (*KIF1A*, c.4682G>A, p.Arg1561His; *DYNC1H1*, c.6343A>G, P.Lys2115Glu) inherited from the healthy one of the parents (Figure 1C).

The variant resulting in Ser743Phe occurred in the cyclic nucleotide monophosphate-binding homology (CNBH) domain adjacent to the C terminus of the putative protein product (Figure 1D). We also found that c.2228C>T in *CNNM2* was a rare variant and not present in any publicly available databases, including the Exome Aggregation Consortium database, ESP6500 database, 1,000 Genomes Project, cg69 database and Genome Aggregation database. In addition, the novel variant has not been reported in the published literature previously and were clearly predicted to be functionally deleterious by the prediction tool ClinPred with a score of 0.741, SIFT with a score of 0.007 and CADD with a score of 23.1, although PolyPhen-2 tool showed benign prediction with a score of 0.013. Therefore, based on the information above and the international guidelines of the American College of Medical Genetics (ACMG) Laboratory Practice Committee Working Group, the variant was classified as a likely pathogenic variant and confirmed as a *de novo* variant by parental samples.

## Discussion

Up to now, 99 types of variants in *CNNM2* have been reported (Table 1). Two heterozygous variants in *CNNM2* (c.117delG, p.Ile40SerfsX15; c.1703C>T, p.Thr568Ile) occurred in four patients of two unrelated families (Stuiver et al., 2011). One variant in CNNM2 located in extracellular domains, the other located in Bateman modules. And all four patients showed hypomagnesemia with a renal defect in Mg^2+^ reabsorption. The other two homozygous variants (c.364G>A, p.Glu122Lys; c.1642G>A, p.Val548Met) (Arjona et al., 2014; Accogli et al., 2019) and 13 heterozygous in *CNNM2* occurred in unrelated families (Kosmicki et al., 2017; Snoeijen-Schouwenaars et al., 2019; Bamhraz et al., 2020; Franken et al., 2021). In 24 cases, different variant domains were listed in Figure 1E. We found DUF21 was the dominant domain (9/24) from the figure. Almost all patients showed hypomagnesemia (except our case and F8 in reference Franken et al., 2021) and varying degrees of intellectual disability. Patients with heterozygous variants showed mild to moderate intellectual disability with impaired motor and language skills, whereas patients with homozygous variants showed severe intellectual disability with very limited motor skills and no verbal. Besides, 16 patients showed a seizure as the symptom at onset, two patients were diagnosed with myoclonus or anorexia nervosa. Epilepsy in patients with heterozygous variants can be well controlled with antiepileptic drugs such as phenobarbital, valproate and clobazam. On the contrary, patients with homozygous variants showed refractory epilepsy, only one of whom responded to valproate and lamotrigine. Furthermore, patients with homozygous variants presented with brain anomalies and microcephaly. Thus, it can be seen that the severity of the neurological impairment seems to be related to the pattern of inheritance. In the 16 patients, Mg^2+^ supplementation therapy failed to completely correct the hypomagnesemia, meanwhile it cannot control the seizures. So we speculated that hypomagnesemia is not the possible cause of epileptic seizures by the variant of CNNM2.

**Table 1:** Clinical, biochemical and neuroradiological data of patients with CNNM2 variants.

	**Stuiver et al. (** **2011** **)**				**Arjona et al. (** **2014** **)**						**Accogli et al. (2019)**	**Bamhraz et al. (2020)**	**Our case**
Patient	II.3	III.2	I.2	II.1	F1.1	F1.2	F2.1	F3.1	F4.1	F5.1	III.1	NA	proband
Gender	M	F	F	M	M	F	F	F	M	F	M	F	F
Ethnicity	Dutch	Dutch	Czech Republic	Czech Republic	Serbian	Serbian	German	German	German	Polish	Moroccan	NA	Chinese
Age onset	15 years	1 year	NA	16 years	1 day	6 days	7 months	1 year	4 months	1 6years	1 day	15 years	3 years
Symptoms at onset	Muscle spasms Headache, palpitations	Muscle spasms, stuttering, loss of consciousness	Symptomless	Weakness, Vertigo, headache	Seizure	Seizure	Seizure	Seizure, paresthesia	Seizure	Myoclonus, paresthesia	Seizure	Anorexia nervosa	Seizure
Initial serum Mg^2+^(mmol/L)	0.46	0.51	0.52	0.36	0.5	0.5	0.56	0.44	0.5	0.66	0.38	0.53	0.74
Mg^2+^ after supplementation (mmol/L)	NA	0.58	NA^*^	0.61	0.66	0.54	0.56	0.53	0.68	NA	0.49	0.56	0.78^*^
Epilepsy treatment	N	N	N	N	Response to valproate and Lamotrigine	Resistance to Valproate and Levertiracetam	Response to Phenobarbital	Response to Valproate	Response to Clobazam	NA	Drug-resistant	N	Drug-resistant
Intellectual disability	N	N	N	N	Severe	Severe	Moderate	Moderate	Moderate	Mild	Severe	Mild	Mild
Language impairment	N	N	N	N	N	N	ELD	ELD	ELD	NA	N	NA	ELD
Motor skills impairment	N	N	N	N	Very limited	Very limited	Impaired	Impaired	Impaired	NA	Very limited	NA	Delayed walking
malformation	N	N	N	N	Microcephaly	Microcephaly	NA	NA	NA	NA	wide mouth, pectus xcavatum, Microcephaly	N	pectus excavatum
Abnormal brain development	NA	NA	NA	NA	Widened outer cerebrospinal liquor space, myelination defects, opercularization defect	NA	N	N	N	NA	Global reduction of white matter, cerebral cortical atrophy	-	NA
variant (DNA level)	c.117delG	c.117delG	c.1703C > T	c.1703C > T	c.364G > A	c.364G > A	c.1069G > A	c.806C > G	c.1069G > A	c.988C > T	c.1642G > A	NA	c.2228C > T
variant (Protein level)	p.Ile40SerfsX15	p.Ile40SerfsX15	p.Thr568Ile	p.Thr568Ile	p.Glu122Lys	p.Glu122Lys	p.Glu357Lys	p.Ser269Trp	p.Glu357Lys	p.Leu330Phe	p.Val548Met	p.Arg366Pro	p.Ser743Phe
variant domain	Extracellular	Extracellular	Bateman module	Bateman module	Extracellular	Extracellular	DUF21	DUF21	DUF21	DUF21	Bateman module	DUF21	CNBH
Zygosity	Heterozygous	Heterozygous	Heterozygous	Heterozygous	Homozygous	Homozygous	Heterozygous	Heterozygous	Heterozygous	Heterozygous	Homozygous	Heterozygous	Heterozygous
	**Kosmicki et al. (** **2017** **)**	**Snoeijen-Schouwenaars et al. (** **2019** **)3**	**Franken et al. (** **2021** **)**										
Patient	NA	NA	F1	F2	F3	F4		F5	F6	F7	F8	F9	
Gender	NA	NA	F	M	M	M		M	M	F	F	F	
Ethnicity	NA	Netherlands	Caucasian	Sub-Saharan African	Caucasian	Caucasian		Sub-Saharan African	Caucasian	Caucasian	Caucasian	Caucasian	
Age onset	NA	NA	6 years	3 months	2 years	3 years		8 months	13 months	16 years	8 months	1–1.5 year	
Symptoms at onset	Epilepsy, autism spectrum disorder	Autism spectrum disorder, Intellectual disability, seizures and epilepsy	seizures	seizures	seizures	N		seizures	seizures	seizures	N	seizures, autism spectrum disorder	
Initial serum Mg^2+^(mmol/L)	NA	NA	0.63	0.57	0.45	0.48		0.5	0.54	0.49	0.72	0.57	
Serum Mg^2+^ after supplementation (mmol/L)	NA	NA	0.65	NA	0.53-0.66	NA		0.51	0.52	0.58	0.7	0.69	
Epilepsy treatment	NA	NA	NA	NA	NA	N		NA	NA	NA	N	NA	
Intellectual disability	NA	NA	Y	Y	N	Y		Y	Y	Y	Y	Y	
Language impairment	NA	NA	ELD	N	ELD	N		ELD	ELD	NA	ELD	ELD	
Motor skills impairment	NA	NA	Y	Y	Y	N		Y	Y	N	Y	N	
Malformation	NA	NA	NA	NA	NA	NA		NA	NA	NA	NA	NA	
Abnormal brain development	NA	NA	N	N	N	N		N	N	N	N	slightly hyperintense white matter	
Mutation (DNA level)	c.154C>T	c.2291G>A	Del ex1-4	Del ex3-8	c.143T>C	c.942C>G		c.961_963del	c.970G>A	c.1253T>C	c.2384C>T	c.2389C>T	
Mutation (Protein level)	p.Leu52Phe	p.Arg764Gln	NA	NA	p.Leu48Pro	p.Tyr314^*^		p.Leu321del	p.Val324Met	p.Leu418Pro	p.Ser795Leu	p.Arg797^*^	
Mutational domain	Extracellular	CNBH	NA	NA	signal peptide domain	DUF21		DUF21	DUF21	DUF21	CNBH	CNBH	
Zygosity	NA	NA	Heterozygous	Heterozygous	Heterozygous	Heterozygous		Heterozygous	Heterozygous	Heterozygous	Heterozygous	Heterozygous	

*NA, not available; ELD, expressive language disorder; F, female; M, male*.

* *no Mg^2+^ supplementation; Y, yes; N, no*.

Our patient showed an intractable epilepsy, characterized by focal seizures. Seizure activity can not be completely controlled despite antiepileptic treatment with oxcarbazepine, valproic acid, topiramate and lacosamide. She was also diagnosed with a mild intellectual disability and a delay in walking and speaking, poor verbal and reading skills and a head circumference below age expectations. Interestingly, she showed no hypomagnesemia, but the serum magnesium was low, which can not be excluded from deficient nutrient intake. In reference Franken et al. (2021), F8 also presented with normal serum magnesium via taking Mg^2+^ supplementation. The two patients had something in common: a de novo mutation in CNBH domain, manifested as seizures, ID and different degrees of language expression disorder. The phenotype of all CNNM2 cases and our patient is summarized in Table 1.

Mg^2+^ is mostly absorbed in the intestinal epithelia, and complemented by a reabsorption in the kidney. Serum Mg^2+^ levels are fine-tuned by transcellular Mg^2+^ reabsorption which occurs in the distal renal tubule (Corral-Rodriguez et al., 2014). As a transporter of Mg^2+^, CNNM2 is highly expressed in the basolateral membrane of the distal renal tubule, mediating the reabsorption of Mg^2+^. CNNMs show a modular structure, which contains an extracellular N-terminal domain, a DUF21 domain that is composed of four transmembrane α-helices, a CNBH domain, a Bateman module including two consecutive cystathionine β-synthase (CBS) motifs (CBS1 and CBS2) and linkers of different length for connection (Corral-Rodriguez et al., 2014; Gimenez-Mascarell et al., 2019). The Bateman module of CNNMs forms a disc-like symmetric dimer called the CBS module (Mahmood et al., 2009), which represents the most conserved region and the most extensively studied region of CNNM proteins. The CBS module interacts with nucleotides in a Mg^2+^ dependent manner and results in its own change in conformation from a “twisted” structure toward a “flat” disc-like state, which might account for the rapid transport of Mg^2+^ by CNNMs. Interestingly, the pathological variant T568I in CNNM2, found in patients who suffer from familial dominant hypomagnesaemia, locks the CBS modules in the “flat” conformation and might hinder it from returning to the “twisted” conformation, makes the CBS modules nonfunctional (Hirata et al., 2014; Gimenez-Mascarell et al., 2019). The failure of the CBS modules may account for the impaired transport of Mg^2+^, leading to hypomagnesemia.

Compared with the CBS domain, the CNBH domain is less well known. Except for a large variable loop, CNBH domains of CNNMs show a great conservation among isoforms (Chen et al., 2018). The CNBH domain of CNNMs resembles numerous cyclic nucleotide-binding domains of cyclic nucleotide-gated channels structurally. The role and exact mechanism of the CNBH domain of CNNMs in Mg^2+^ transport remain uncertain. Similar to the CBS domains, the cyclic nucleotide-binding domains of cyclic nucleotide monophosphate (cNMP)-dependent kinases and ion channels are known to be capable of interacting with cyclic nucleotides and then having structural modifications (Shabb and Corbin, 1992). Interestingly, the nuclear magnetic resonance (NMR) and thermal shift assays (TSA) have confirmed that the CNBH domain of CNNMs lacks the ability of binding cyclic nucleotides such as cAMP and cGMP. Instead, the function of CNBH domains may be dimerization, regulating the activity of Mg^2+^ efflux in the basolateral membrane of epithelial cells and mediating the absorption of Mg^2+^ in a cyclic nucleotide-independent manner. Nevertheless, variants that prevent CNBH dimerization had divergent influences in the cellular Mg^2+^ efflux, one showed obviously efflux impairment whereas another manifested close to efflux of wild-type (Chen et al., 2018). It can be speculated that the dimerization is crucial but not necessary for Mg^2+^ efflux and some variants in CNBH domain may not impair the Mg^2+^ efflux activity of CNNMs significantly. The pathogenic variant of our case is located in CNBH domain, which is the rarely variant site in the CNBH domain of CNNM2.

In CNNM2, the CNBH module exists as homodimers and associates each other in dimers. Gehring et al. elucidated the integral fold of the CNBH module of CNNM2 at 2.6 Å resolution, which showed a central eight-stranded antiparallel β roll that has two α-helixes in the N-terminal and an α-helix in the C-terminal. Thereinto, residues 724 to 767 of CNNM2 were deleted because they were hardly conserved between isoforms and were not predicted to form a regular secondary structure (Chen et al., 2018). However, the pathogenic variant Ser743Phe of our case is located in residues 724 to 767 of CNNM2. And it is reasonable to conjecture that the special phenotype (no hypomagnesemia) in our case results from the special location of the variant. Of course, the presence of a innocuous polymorphism can not be absolutely ruled out. But the clinical phenotype with mild intellectual disability and intractable epilepsy supports a partial loss of CNNM2 function due to the p.Ser743Phe variant.

For patients with heterozygous or homozygous variant in CNNM2, seizures and hypomagnesemia constituted the major symptom. Low serum Mg^2+^ is related to a series of neurological conditions, including epilepsy. In the nervous system, N-methyl-D-aspartate (NMDA) receptors, which play an important role in excitatory synaptic transmission, are directly inhibited by the extracellular Mg^2+^concentration. Whereas GABAA receptors, whose function is directly excited by Mg^2+^concentration, have an inhibitory function (Nowak et al., 1984; Paoletti et al., 2013). When the concentration of Mg^2+^ is low, NMDA receptors are overexcited and GABAA receptors become less excited, making neurons hyperexcitability and leading to epileptic activity (Moykkynen et al., 2001). Therefore, it is undeniable that severely Mg^2+^ deficiency plays a stimulative role in the seizures development. However, some authors thought that variants in CNNM2 were the reason why intellectual disability and seizures occurred in patients with hypomagnesemia. Arjona et al. injected morpholino (MO) into zebrafish embryos to block the translation of early expressed zebrafish *CNNM2* paralogues. At non-lethal doses of MO, knockdown of *CNNM2* paralogues lead to morphological phenotypes characterized by accumulation of cerebrospinal fluid, motor neuronal phenotype characterized by increased embryonic spontaneous contractions and cerebral development defect. All phenotypes were rescued by co-injection with wild-type *Cnnm2* cRNA of the mouse rather than co-injection with mutant *CNNM2* cRNA or exposure to the media with high Mg^2+^ concentrations. Interestingly, “co-injection with mutant *CNNM2* cRNA even worsened motor neuronal phenotype by increasing the number of embryonic spontaneous contractions significantly” (Arjona et al., 2014). It can be proven that *CNNM2* gene is crucial for cerebral development and neurological function. And in patients, seizure activity continued despite normal serum Mg^2+^ levels, which prompted that it might be a genuine brain function disturbance caused by impaired CNNM2 but not hypomagnesemia. This is the reason that oral Mg^2+^ therapy failed to control seizures.

Our patient showed a mild intellectual disability, with a *de novo* heterozygous missense *CNNM2* variant. Reviewing the literature (Stuiver et al., 2011; Arjona et al., 2014; Accogli et al., 2019; Bamhraz et al., 2020; Franken et al., 2021), we found that severe intellectual disability all occurred in patients with homozygous missense variants. Hence, it can be speculated that homozygous variants significantly affect the function of CNNM2, leading to severe phenotypes. In contrast, the heterozygous state of their parents may not so sufficient to cause functional loss, so the Mg^2+^ homeostasis was maintained. Compared to patients with homozygous variants in the extracellular domain, those with homozygous variants in the CBS domain had more severe hypomagnesemia and more intractable epilepsy. In summary, these results support the viewpoint that the severity of the disease correlates with the number of variant alleles and the damaged protein domains.

## Concluding Remarks

In summary, we have presented a *de novo* heterozygous *CNNM2* variant, and the CNBH domain of *CNNM2* possibly plays a limited role in Mg^2+^ transport but a significant role in neural development. So we speculated that neurological phenotypes such as intellectual disability and seizures may be purely caused by *CNNM2* variants, and the number of variant alleles and the changed protein domains correlates with the severity of the disease. *CNNM2* variants should also be considered in patients with epilepsy and intellectual disability, even no hypomagnesemia.

## Data Availability Statement

The datasets presented in this study can be found in online repositories. The names of the repository/repositories and accession number(s) can be found in the article/supplementary material.

## Ethics Statement

Written informed consent was obtained from the individual(s), and minor(s)' legal guardian/next of kin, for the publication of any potentially identifiable images or data included in this article.

## Author Contributions

XL, SB and WW collected data and wrote the manuscript. XS, YH, and FL reviewed the article. QZ and FZ draw the table and the graph. ZL directed and revised the manuscript. All authors contributed to the article and approved the submitted version.

## Conflict of Interest

The authors declare that the research was conducted in the absence of any commercial or financial relationships that could be construed as a potential conflict of interest.

## Publisher's Note

All claims expressed in this article are solely those of the authors and do not necessarily represent those of their affiliated organizations, or those of the publisher, the editors and the reviewers. Any product that may be evaluated in this article, or claim that may be made by its manufacturer, is not guaranteed or endorsed by the publisher.
